# Supplementary material for: Experience-dependent modulation of collective behavior in larval zebrafish
Source: bioRxiv. 2024 Aug 5:2024.08.02.606403. Preprint. [Version 1] doi: 10.1101/2024.08.02.606403 (PMC11326175; doi:10.1101/2024.08.02.606403)
Supplement: Supplement 5 [file NIHPP2024.08.02.606403v1-supplement-5.pdf]

bioRxiv preprint doi: https://doi.org/10.1101/2024.08.02.606403; this version posted August 5, 2024. The copyright holder for this preprint (which was not certified by peer review) is the author/funder, who has granted bioRxiv a license to display the preprint in perpetuity. It is made available under aCC-BY 4.0 International license.

Supplementary figures:

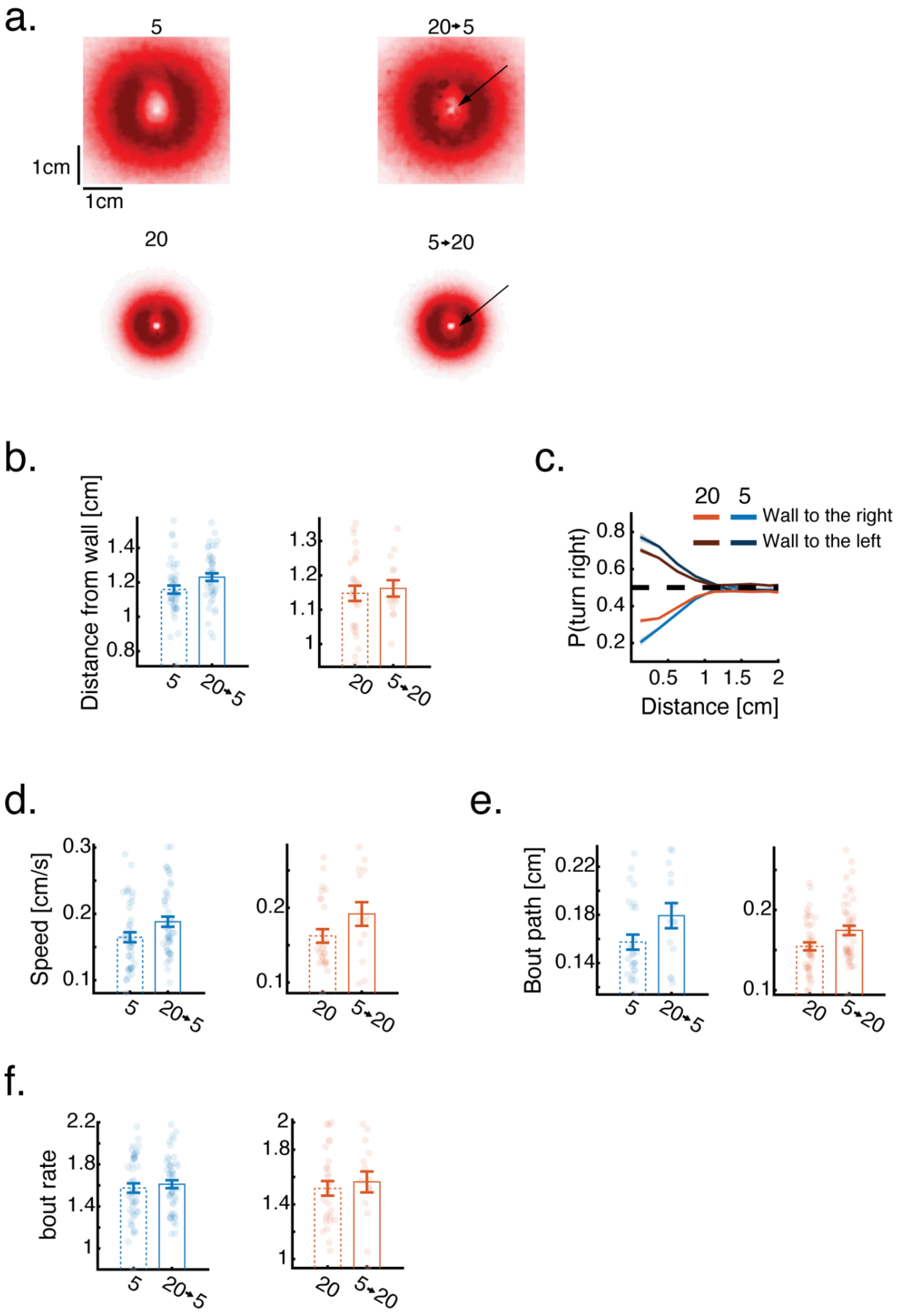

bioRxiv preprint doi: https://doi.org/10.1101/2024.08.02.606403; this version posted August 5, 2024. The copyright holder for this preprint (which was not certified by peer review) is the author/funder, who has granted bioRxiv a license to display the preprint in perpetuity. It is made available under aCC-BY 4.0 International license.

Figure S1 - Previous social experience modulates collective behavior. a. Density maps depicting the 2d positions of nearest neighbor fish with respect to a focal fish situated at the center of the map pointing north. Maps are calculated from fish swimming in low (top row) and high (bottom row) neighbor densities that were either exposed to the opposite neighbor density in the past (right column) or to a similar density. Arrows point to areas of marked differences in density maps due to previous social experiences. **b.** Average wall distance of groups (Wall $distance_{5}$=1.16$\pm$0.16, Wall $distance_{20\rightarrow5}$=1.23$\pm$ 0.15 [mean$\pm$SD], p=0.0143, Wilcoxson's rank sum test; Wall $distance_{20}$=1.14$\pm$0.12, Wall $distance_{5\rightarrow20}$=1.16$\pm$0.09 [mean$\pm$SD], p=0.77, Wilcoxson's rank sum test). **c.** Probability to turn right as a function of distance and direction (left or right) to the nearest wall for groups of 5 (blue colors) and 20 (red colors) fish. Lines represent mean turning probability calculated as the fraction of right turns out of all turns in 0.25 cm bins. Shaded areas are SEM. **d.** Average swimming speed of fish in groups ($Speed_{5}$=0.16$\pm$0.05, $Speed_{20\rightarrow5}$=0.19$\pm$0.05 [mean$\pm$SD] p=0.0287, Wilcoxson's rank sum test; $Speed_{20}$=0.16$\pm$0.05, $Speed_{5\rightarrow20}$=0.19$\pm$0.06 [mean$\pm$SD], p=0.11, Wilcoxson's rank sum test). **e.** Average path traveled in a bout of fish in groups (Bout $path_{5}$=0.15$\pm$0.03, Bout $path_{20\rightarrow5}$=0.17$\pm$0.04 [mean$\pm$SD], p=0.0157 Wilcoxson's rank sum test; Bout $path_{20}$=0.16$\pm$0.03, Bout $path_{5\rightarrow20}$=0.18$\pm$0.03 [mean$\pm$SD], p=0.0962, Wilcoxson's rank sum test) **f.** Average bout rate of fish in groups (Bout $rate_{5}$=1.57$\pm$0.3, Bout $rate_{20\rightarrow5}$=1.61$\pm$0.26 [mean$\pm$SD], p=0.71 Wilcoxson's rank sum test; Bout $rate_{20}$=1.51$\pm$0.27, Bout $rate_{5\rightarrow20}$=1.56$\pm$0.29 [mean$\pm$SD], p=0.42, Wilcoxson's rank sum test). In panels (b,d-f) Dots represent individual groups, error bars are mean$\pm$SEM.

bioRxiv preprint doi: https://doi.org/10.1101/2024.08.02.606403; this version posted August 5, 2024. The copyright holder for this preprint (which was not certified by peer review) is the author/funder, who has granted bioRxiv a license to display the preprint in perpetuity. It is made available under aCC-BY 4.0 International license.

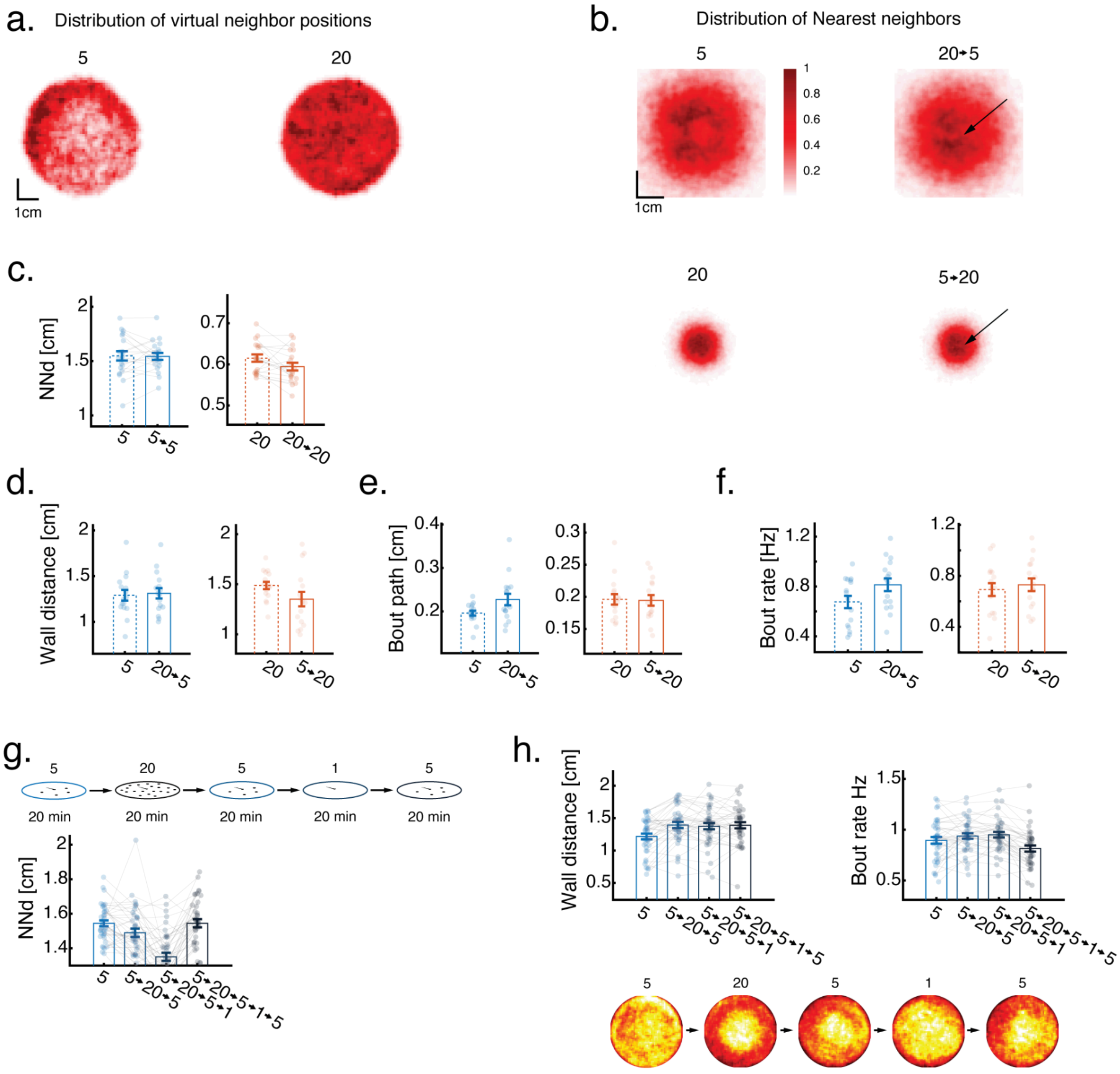

Figure S2 - Virtual reality reveals the temporal characteristics of experience dependent modulation. a. Position density of real fish trajectories used as stimulus fish in VR experiments. Trajectories are characterized by some naturally occurring inhomogeneities. Using simulated trajectories instead of real fish trajectories, did not qualitatively change our findings. **b.** Density maps depicting the 2d positions of virtual nearest neighbors with respect to a focal fish situated at the center of the map pointing north. Maps are calculated from fish swimming in low (top row) and high (bottom row) virtual neighbor densities that were either exposed to the opposite neighbor density in the past (right column) or to a similar density (left column). Arrows point to areas of marked differences in density maps due to previous social experiences. **c.** Average nearest neighbor distance of real fish that did not experience a change in virtual neighbor density (NN_5=1.547±0.19, $NN_{5\to5}$=1.544±0.14 [mean± SD], p=1 Wilcoxon's signed rank; NN_{20}=0.615±0.04, $NN_{20\to20}$=0.594±0.04 [mean±SD], p=0.02 Wilcoxon's signed rank). **d.** Average wall distance of real fish (Wall distance$_5$=1.29±0.23, Wall distance$_{20\to5}$=1.31±0.22 [mean±SD], p=0.9 Wilcoxon's rank sum; Wall distance$_{20}$=1.49±0.15, Wall

bioRxiv preprint doi: https://doi.org/10.1101/2024.08.02.606403; this version posted August 5, 2024. The copyright holder for this preprint (which was not certified by peer review) is the author/funder, who has granted bioRxiv a license to display the preprint in perpetuity. It is made available under aCC-BY 4.0 International license.

distance$_{5\rightarrow20}$=1.35$\pm$0.29 [mean$\pm$SD], p=0.057 Wilcoxson's rank sum). **e.** Average path traveled within a bout of real fish (Bout path$_5$=0.2$\pm$0.02, NN$_{20\rightarrow5}$=0.22$\pm$0.05 [mean$\pm$SD], p=0.067 Wilcoxson's rank sum; Bout path$_{20}$=0.2$\pm$0.03, NN$_{5\rightarrow20}$=0.2$\pm$0.03 [mean$\pm$SD], p=1 Wilcoxson's rank sum). **f.** Average bout rate of real fish (Bout rate$_5$=0.67$\pm$0.2, NN$_{20\rightarrow5}$=0.82$\pm$0.2 [mean$\pm$SD], p=0.062 Wilcoxson's rank sum; Bout rate$_{20}$=0.7$\pm$0.2, NN$_{5\rightarrow20}$=0.73$\pm$0.2 [mean$\pm$SD], p=0.777 Wilcoxson's rank sum). **g.** Nearest neighbor distance of real fish before and after exposure to high density and after subsequent exposure to 0 virtual neighbors (NN$_5$=1.54$\pm$0.1, NN$_{5\rightarrow20\rightarrow5}$=1.49$\pm$0.15 [mean$\pm$SD], p=0.005 Wilcoxson's signed rank; NN$_5$=1.54$\pm$0.1, NN$_{5\rightarrow20\rightarrow5\rightarrow0\rightarrow5}$=1.54$\pm$0.15 [mean$\pm$SD], p=0.7 Wilcoxson's signed rank). **h. Top:** Wall distance, bout rate and position density of real fish before and after exposure to high density and after subsequent exposure to 0 virtual neighbors (Wall distance$_5$=1.22$\pm$0.28, Wall distance$_{5\rightarrow20\rightarrow5}$=1.39$\pm$0.29 [mean$\pm$SD], p=0.0002 Wilcoxson's signed rank; Wall distance$_{5\rightarrow20\rightarrow5}$=1.39$\pm$0.29, Wall distance$_{5\rightarrow20\rightarrow5\rightarrow0\rightarrow5}$=1.39$\pm$0.29 [mean$\pm$SD], p=0.81 Wilcoxson's signed rank). **Bottom:** position density maps of real fish in the 5 subsequent experimental conditions. In panels (c-h) dots represent fish, lines connect data from same fish, error bars are mean$\pm$SEM.

bioRxiv preprint doi: https://doi.org/10.1101/2024.08.02.606403; this version posted August 5, 2024. The copyright holder for this preprint (which was not certified by peer review) is the author/funder, who has granted bioRxiv a license to display the preprint in perpetuity. It is made available under aCC-BY 4.0 International license.

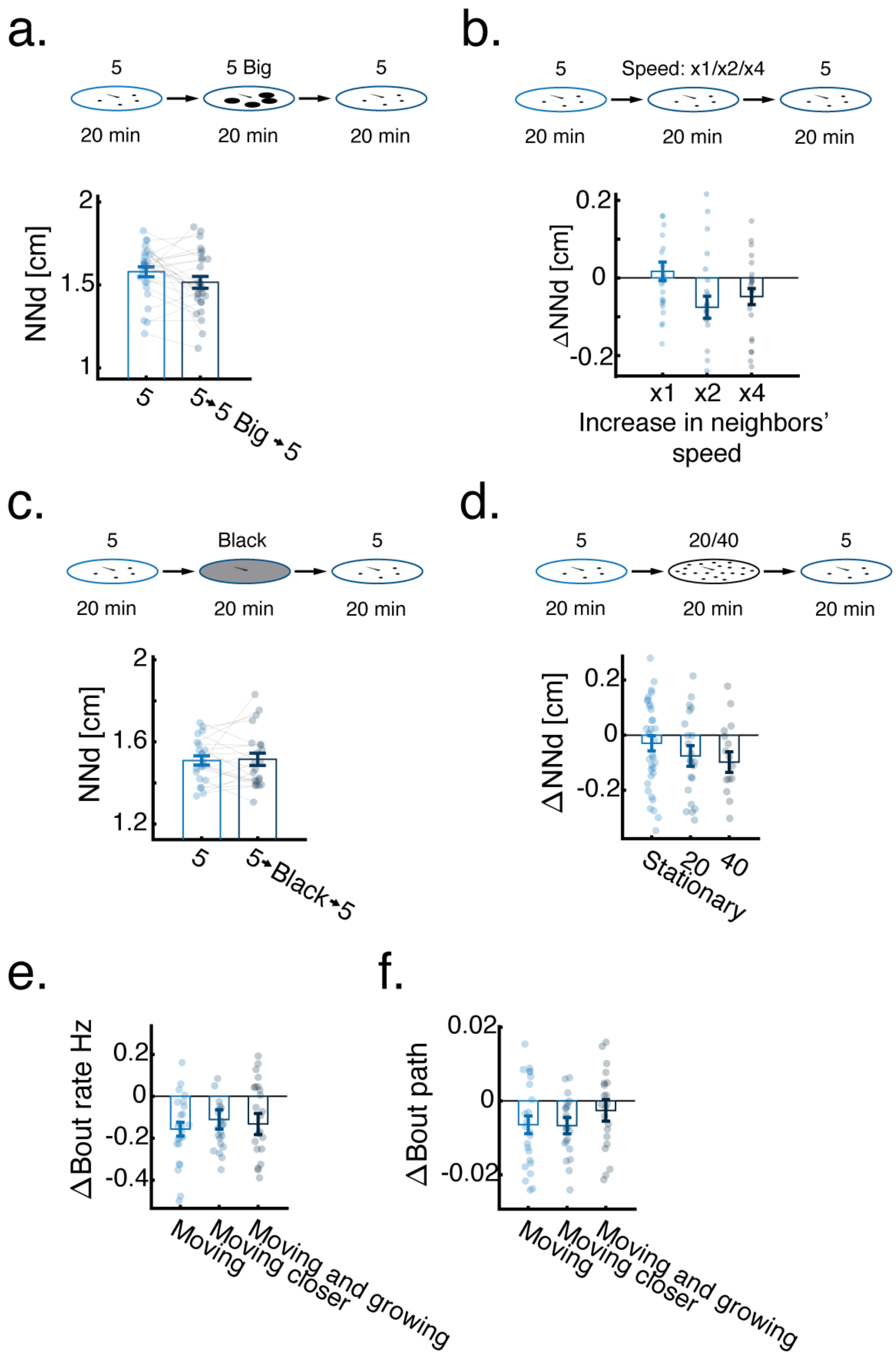

Figure S3 - Persistent changes in retinal occupancy drive experience-dependent modulation of collective behavior. a. Average nearest neighbor distance of fish swimming in a low density group before and after 20 min of exposure to the same number of virtual neighbors, but with a larger size - $r_{large}=4 \cdot r_{small}$ ($NN_5=1.58 \pm 0.15$, $NN_{5\to5\ big\to5}=1.51 \pm 0.18$ [mean±SD], p = 0.067 Wilcoxon signed rank). **b.** Average change (after-before) in nearest neighbor distance of fish swimming in a low density group after 20 min of exposure to the same density of neighbors swimming in different speeds (ΔNN_{speed}

bioRxiv preprint doi: https://doi.org/10.1101/2024.08.02.606403; this version posted August 5, 2024. The copyright holder for this preprint (which was not certified by peer review) is the author/funder, who has granted bioRxiv a license to display the preprint in perpetuity. It is made available under aCC-BY 4.0 International license.

$_{x1}$=0.017±0.12, $\Delta NN_{speed\ x2}$=-0.076±0.0.14, $\Delta NN_{speed\ x4}$=-0.048±0.1 [mean±SD], $p_{speed\ x1}$=0.8, $p_{speed\ x2}$=0.013, $p_{speed\ x4}$=0.032 Wilcoxson signed rank). **c.** Average nearest neighbor distance of fish swimming in a low density group before and after exposure to 20 min of a black whole-field background (NN_5=1.51±0.1, $NN_{5\rightarrow black\rightarrow 5}$=1.515±0.14 [mean±SD], p = 0.98 Wilcoxon signed rank). **d.** Average change (after-before) in nearest neighbor distance of fish swimming in a low density group after exposure to 20 min of different high densities of neighbors ($\Delta NN_{stationary\ 20}$=0.032±0.13, ΔNN_{20}=-0.075±0.18, ΔNN_{40}=-0.1±0.15 [mean±SD], $p_{stationary\ 20}$=0.387, p_{20}=0.059, p_{40}=0.03 Wilcoxson signed rank). **e.** Average change (after-before) in bout rate of fish swimming in a low density group after exposure to 20 min of high density of neighbors with different motion statistics (Δbout $rate_{moving}$=-0.16±0.16, Δbout $rate_{moving\ closer}$=-0.11±0.22, Δbout $rate_{moving\ and\ growing}$=-0.13±0.24 [mean±SD], p_{moving}=0.0003, $p_{moving\ closer}$=0.0072, $p_{moving\ and\ growing}$=0.015 Wilcoxson signed rank). **f.** Average change (after-before) in bout path of fish swimming in a low density group after exposure to 20 min of high density of neighbors with different motion statistics (Δbout $path_{moving}$=-0.0064±0.012, Δbout $path_{moving\ closer}$=-0.0067±0.011, Δbout $path_{moving\ and\ growing}$=-0.0026±0.014 [mean±SD], p_{moving}=0.018, $p_{moving\ closer}$=0.0033, $p_{moving\ and\ growing}$=0.465 Wilcoxson signed rank). In panels (a-f) dots represent fish, error bars are mean±SEM.

bioRxiv preprint doi: https://doi.org/10.1101/2024.08.02.606403; this version posted August 5, 2024. The copyright holder for this preprint (which was not certified by peer review) is the author/funder, who has granted bioRxiv a license to display the preprint in perpetuity. It is made available under aCC-BY 4.0 International license.

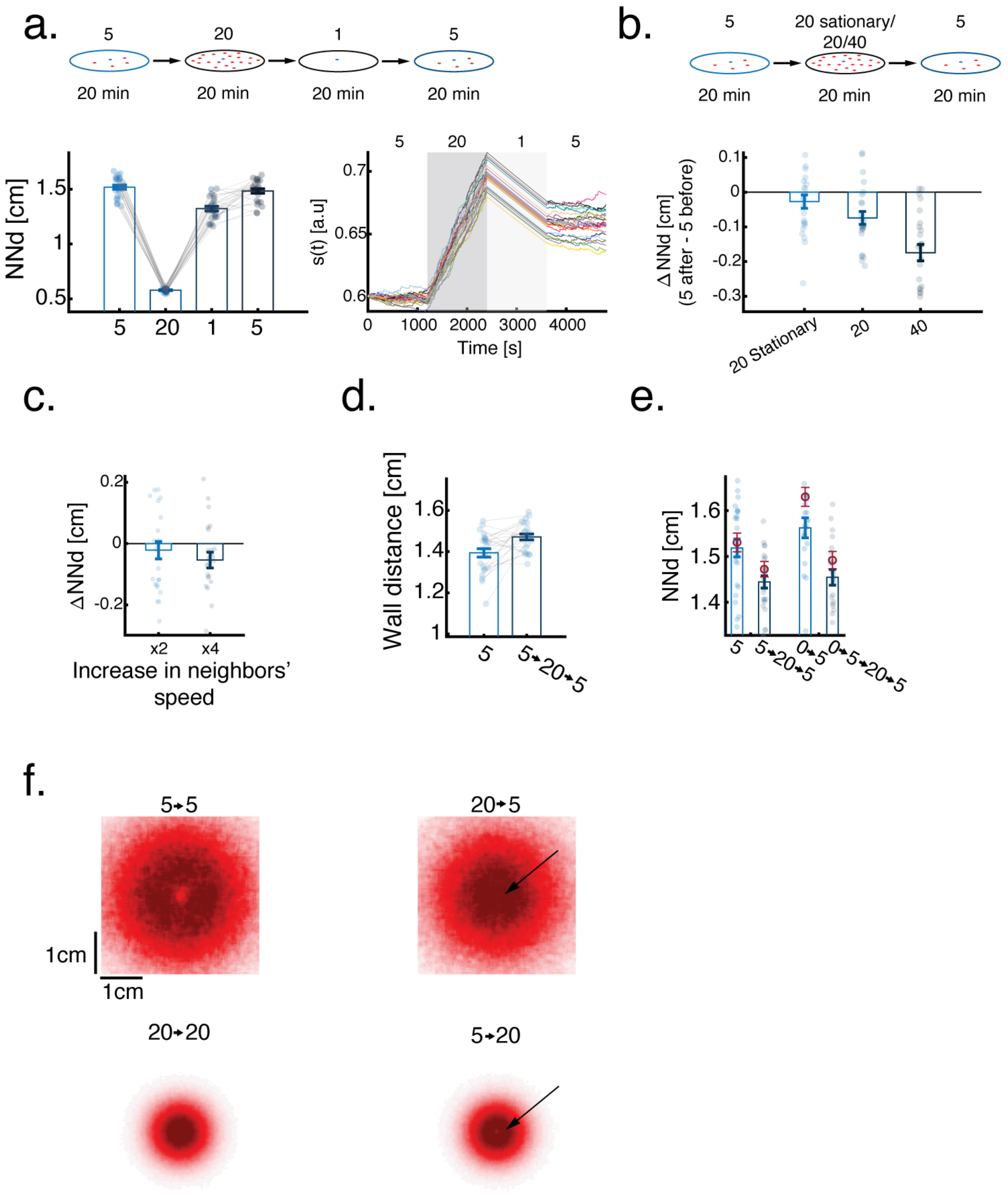

Figure S4 - Models accurately predict experience dependent modulation of collective behavior. **a. Left:** Nearest neighbor distances of simulated agents swimming in different densities of virtual neighbors starting from low (4 neighbors), switching to high (19 neighbors), 0 neighbors and returning to low again. Dots represent different simulated agents (N=24). **Right:** Value of the internal state variable $s(t)$ over time for the same agents shown on the left. Different colors represent different

bioRxiv preprint doi: https://doi.org/10.1101/2024.08.02.606403; this version posted August 5, 2024. The copyright holder for this preprint (which was not certified by peer review) is the author/funder, who has granted bioRxiv a license to display the preprint in perpetuity. It is made available under aCC-BY 4.0 International license.

simulated agents responding to the same neighbor stimuli. $s(t)$ increases sharply with the switch to high density and decays more slowly with the switch to 0 neighbors. After switching back to low density it remains stable for the 20 minutes period. **b.** Average change (after-before) in nearest neighbor distance of fish swimming in a low density group after exposure to 20 min of different high densities of neighbors. **c.** Average change (after-before) in nearest neighbor distance of agents swimming in a low density group after exposure to a low density of virtual neighbors swimming at different speeds. **d.** Average wall distance of agents swimming in a low density group before and after exposure to a high density of virtual neighbors. **e.** Average nearest neighbor distance of agents swimming in a low density group before (light blue) and after (dark blue) exposure to a high density of virtual neighbors, and with (right) or without (left) pre-exposure to 0 neighbors. Dark red circles and error bars represent mean± SEM of the real fish data from similar experiments. **f.** Density maps depicting the 2d positions of nearest neighbors with respect to a focal simulated agent situated at the center of the map pointing north. Maps are calculated for agents swimming in low (top row) and high (bottom row) agent densities that were either exposed to the opposite neighbor density in the past (right column) or to a similar density (left column). Arrows point to areas of marked differences in density maps due to past experience. In panels (b-e) dots represent simulated fish, error bars are mean±SEM and N=24 simulation repetitions.

Movies:

Movie 1: Free swimming behavior of larvae at low and high densities. Groups of 5 and 20 larvae, age 7 dpf, swimming together in a group. Colors represent individual fish; the movie is shown at x4 real speed.

Movie 2: Individual larva swimming at low and high densities of virtual neighbors. A single larval zebrafish, age 7 dpf, swimming in low (4 virtual neighbors) and high (19 virtual neighbors) densities. The movie is shown at x2 real speed.

Movie 3: Individual larva responding to different motion statistics of virtual neighbors. Examples of individual larval zebrafish, age 7 dpf, responding to high densities (19 virtual neighbors) of: (1) stationary neighbors, (2) neighbors moving on concentric circles around the larva (translational motion only), and (3) stationary neighbors with sizes growing and shrinking (looming motion only). The movie is shown at x2 real speed.

Movie 4: Individual larva responding to different motion statistics of a single virtual neighbor. Examples of individual larval zebrafish, age 7 dpf, responding to single dot mimicking a neighboring fish that: (1) moves radially around the fish at a constant distance (moving), (2) moves radially around the fish at decreasing distances (moving closer), and (3) moves radially around the fish at a constant distance and increases in size (moving and growing). The movie is shown at x2 real speed.
